# Dynamic Ghrelin and GH serum levels during combined simultaneous arginine clonidine stimulation test in children with dwarfism

**DOI:** 10.1186/s13052-019-0610-5

**Published:** 2019-01-28

**Authors:** Guangzhong Zhou, Rongzeng Du

**Affiliations:** 1 Department of pediatrics, Jiangdu People’s Hospital of Yangzhou, No. 9 Dongfanghong Road, Yangzhou, China; 2 Jiangbin Hospital Affiliated to Jiangsu University, Zhenjiang, China

**Keywords:** Ghrelin, GH, Arginine, Clonidine, Dwarfism

## Abstract

**Background:**

Combined simultaneous arginine clonidine stimulation (CSACS) test represents a more appropriate stimulus to detect Ghrelin, for it does not affect glucose metabolism.

**Methods:**

Fifty prepubertal children with dwarfism were recruited and further classified into normal growth hormone (NGH) and growth hormone deficiency (GHD) group with growth hormone (GH) peak cut-off value of 10 μg/l. In both groups, GH and Ghrelin serum levels were determined after the GH provocation test at 30, 60, and 120 min and the height standard deviation score (SDS) for bone age was measured six months later.

**Results:**

The participants were classified into NGH (*n* = 24) and GHD group (*n* = 26). A decrease in the circulating Ghrelin levels prior to the GH peak was observed in the NGH children, whereas both GH and Ghrelin levels demonstrated a rise in the GHD children. Ghrelin level in GHD group was higher compared with NGH group and the GH peak in GHD group is lower than NGH group. The 6 months CSACS treatment could increase the height SDS in both groups.

**Conclusion:**

Although analogous changes were not detected in GHD group, the inverse correlation between GH and Ghrelin in NGH indicates a negative feedback lying between GH and Ghrelin.

## Background

Dwarfism can be defined as a standing height more than two standard deviations below the average or below the 3rd percentile (for sex and age) [1–3]. Achondroplasia and growth hormone deficiency (GHD) are responsible for the majority of children dwarfism cases. Excluding other serious diseases, the diagnosis of GHD is usually confirmed by a growth hormone (GH) provocation test where different GH secretagogues (insulin, clonidine, glucagon, arginine, and L-dopa) can be chosen to detect GH peak [4, 5]. In order to improve the discrimination, consecutive tests are suggested. It is worth noting that the further consecutive tests can be affected by down-regulated hypothalamic/pituitary axis which makes the diagnosis of GHD a clinical challenge [6].

Ghrelin is a 28 amino-residue peptide hormone produced by ghrelinergic cells in the gastrointestinal tract which is initially considered to have growth hormone-releasing function and can act as the endogenous ligand of the growth hormone secretagogue receptor type 1α. Due to the strong association with glucose metabolism, the utility of glucagon or insulin for GH provocation may be unsuitable to detect Ghrelin.

Clonidine, as an α-adrenergic agonist to be used as an anti-hypertensive drug, does not affect glucose metabolism and could not only inhibit somatostatin to stimulate the release of GH but also increase the secretion of growth hormone releasing hormone. Arginine can inhibit the release of somatostatin. Thus, the combined arginine and clonidine test could represent a more appropriate stimulus to detect Ghrelin. Some research is performed to optimize the sensitivity and specificity of the combined arginine and clonidine test, the detection time course is shortened [7, 8] and the sequential way of administration is changed into simultaneous delivery [9, 10], which show the similar or even better diagnostic accuracy.

In this study, combined simultaneous arginine clonidine stimulation (CSACS) and treatment in children with dwarfism are performed and dynamic Ghrelin and GH serum levels are monitored.

## Methods

### Subjects

Fifty children (27 boys and 23 girls, age between 8 to 12 years) with dwarfism or restricted growth were recruited in this studied from March 2016 to March 2018 in Jiangdu People’s Hospital of Yangzhou. Before entering the study, the children were given a thorough physical examination, biochemical test (renal, liver, and thyroid function), and neurological examination, without serious chronic disease, intrauterine growth retardation, or unremarkable personal medical history. The growth charts were used to calculate standard deviation score (SDS) as previously reported [11, 12]. All the study procedure was approved by the Ethics Committee of the Jiangdu People’s Hospital of Yangzhou and informed written consents were got from guardians of all participants.

### Combined simultaneous arginine clonidine stimulation

CSACS test was performed as previously reported [10]. After overnight fast, an intravenous catheter was administrated in the antecubital vein to infuse Arginine (arginine hydrochloride, 10% solution, Sigma, St. Louis, MO, USA) at a dose of 0.5 g/kg (maximum 30 g, over 30 min) and clonidine (Catapresan, Boehringer, Germany) was administrated orally at a dose of 0.15 mg/m^2^. Blood pressure was strictly monitored during the whole procedure and blood samples were drawn at baseline, 30, 60, and 120 min after the arginine infusion.

GH reserve or the peak GH response was defined biochemically by the peak serum concentration after CSACS. Serum GH level was determined by immunoradiometric assay (IRMA) (Auto Delfia, Wallac Turku, Finland) and the lower detection limit was 0.01 μg/l. The cut-off value was set at 10 μg/l to classify the dwarfism participants into GHD group and the normal growth hormone (NGH) group.

### Ghrelin and IGF-1 assay

A commercially available radioimmunoassay kit (Phoenix Pharmaceuticals, Inc. Belmont, CA, USA) was used to measure the serum level of Ghrelin which used a rabbit polyclonal antibody against octanoylated human Ghrelin and a ^125^I-labeled Ghrelin tracer. Insulin-like growth factor 1 (IGF-1) levels were determined by a fully automated, two-site chemiluminescent immunoassay (Nichols Advantage®, Nichols Institute Diagnostics).

### The height standard deviation score

The effect of CSACS on growth was confirmed by comparison of height standard deviation score (SDS) which showed the number of standard deviations below or above the mean value of the height [13].

### Statistical analyses

A *t*-test was used to assess the differences between GHD and NGH group. Paired samples *t*-test was used to calculate the changes in different time intervals compared with baseline during CSACS.

## Results

### Combined simultaneous arginine clonidine stimulation increase GHD serum ghrelin level

**Fig. 1:**
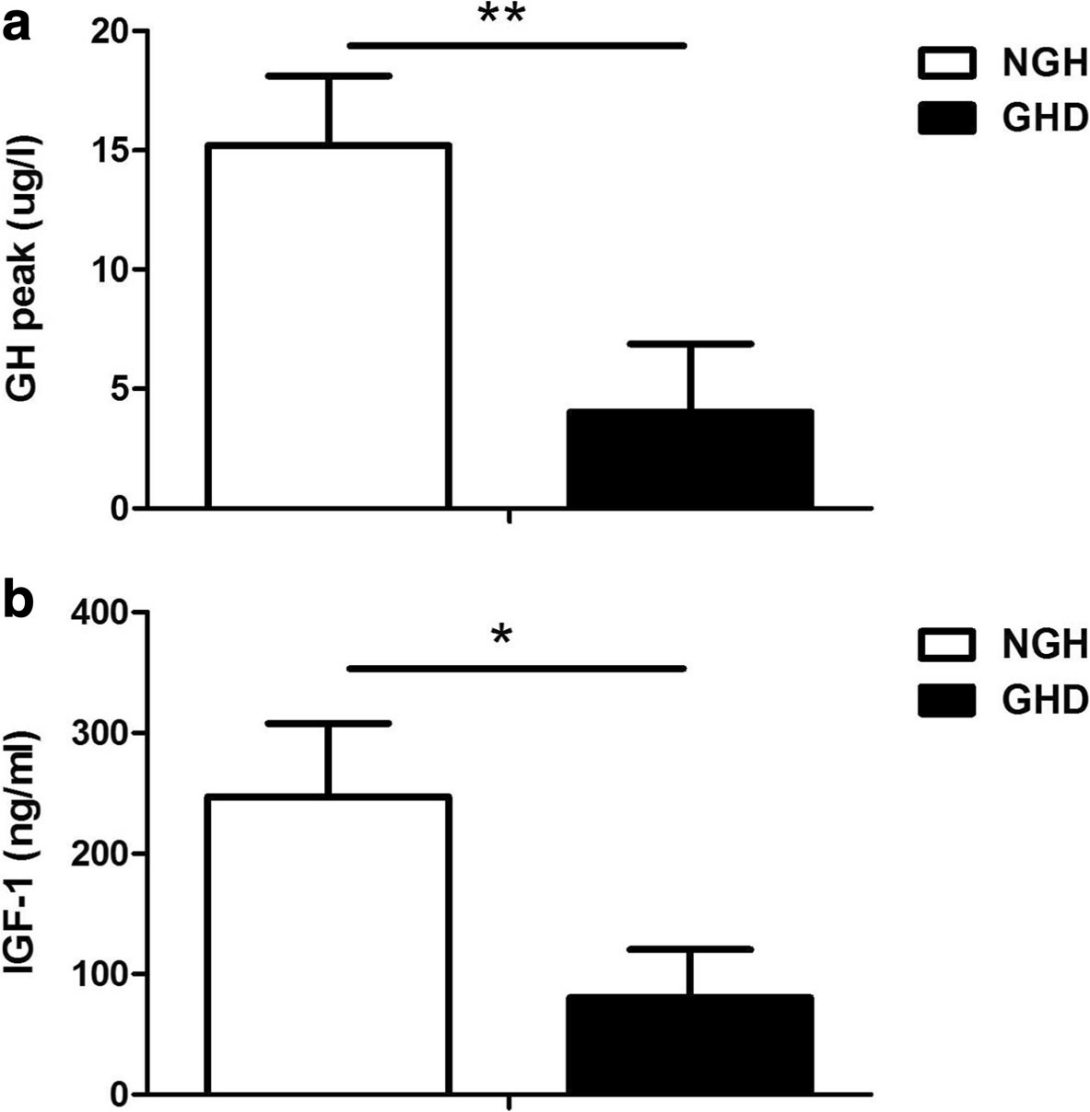
Serum levels of GH peak (**a**) and IGF-1 (**b**) were analyzed in the NGH and GHD groups. Data were shown as mean ± sd, *p* < 0.05*, *p* < 0.01**

50 children diagnosed as dwarfism were further classified into two groups according to cut-off value (peak GH, 10 μg/l): the GHD group (*n* = 26) and the NGH group (*n* = 24). The relevant clinical and biochemical characteristics of the participants were shown in Table 1 and the two groups showed no differences in sex, age, body weight, height, and the baseline Ghrelin levels. When compared with NGH group, GHD group showed decreased serum levels of GH peak (Fig. 1a, *p* < 0.01) and IGF-1 (Fig. 1b, *p* < 0.05).

**Table 1:** Basic characteristics of the study population

	GHD (*n* = 26)	NGH (*n* = 24)	*P* value
Boys/Girls	14/12	13/11	
Age (years)	9.7 ± 0.8	9.6 ± 0.9	0.689
Body weight (kg)	33.6 ± 4.1	32.8 ± 4.5	0.452
Height (cm)	124.2 ± 5.9	124.9 ± 5.2	0.421
GH peak (μg/l)	4.7 ± 3.6	15.2 ± 2.9	0.002
Ghrelin (pmol/l)	652.9 ± 68.2	610.1 ± 65.8	0.398

*GH* Growth hormone, *GHD* growth hormone deficiency, *NGH* normal growth hormone

**Fig. 2:**
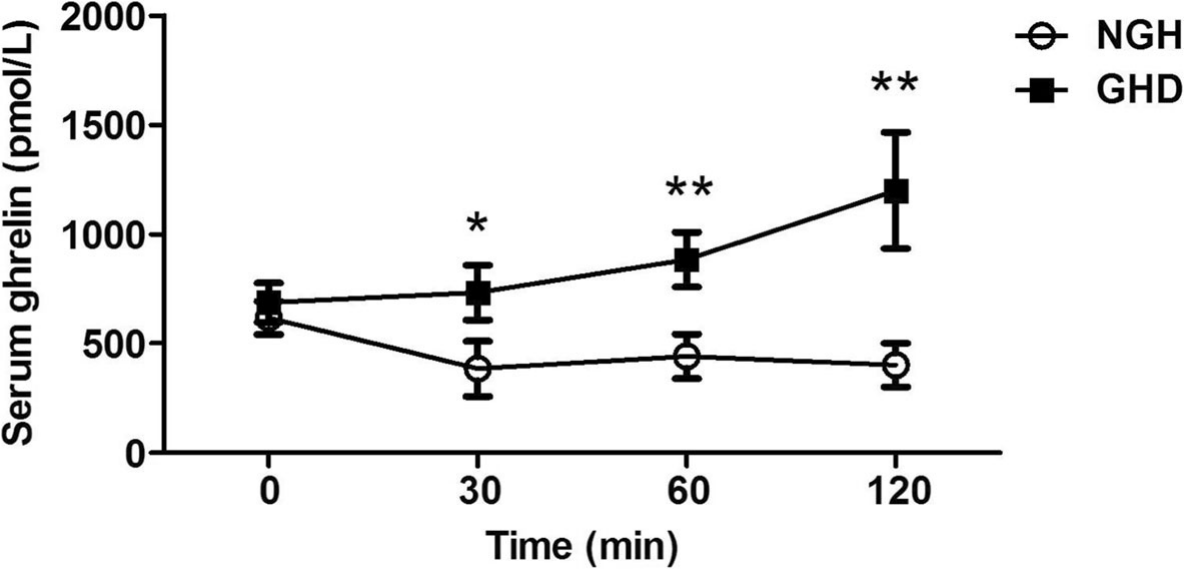
Serum levels of Ghrelin during combined simultaneous arginine clonidine stimulation test were evaluated in the NGH and GHD groups. Data were shown as mean ± sd, p < 0.05*, p < 0.01** when GHD group compared with NGH group

After the CSACS test, participants with GHD demonstrated an increase in serum Ghrelin level at 30, 60, and 120 min compared to baseline, while circulating Ghrelin in the NGH group was lower than baseline. When compared with NGH group, Ghrelin levels in the GHD group were significantly higher at 30 (*p* < 0.05), 60 (*p* < 0.01), and 120 min (p < 0.01) post the CSACS test (Fig. 2).

**Fig. 3:**
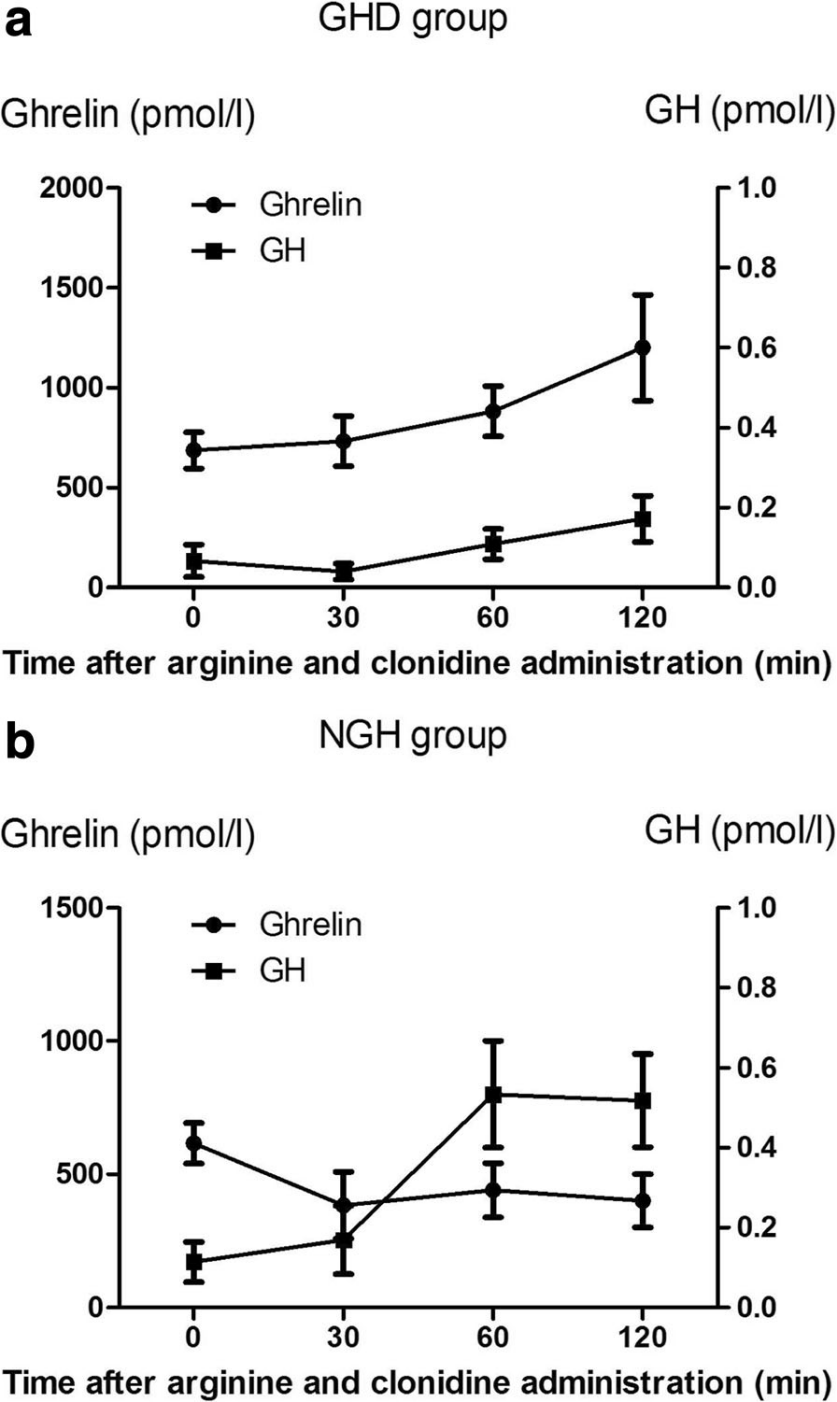
Serum Ghrelin and GH values during combined simultaneous arginine clonidine stimulation test were evaluated in the NGH (**a**) and GHD (**b**) groups. Data were shown as mean ± sd

The mean Ghrelin and GH fluctuations within NGH and GHD groups were demonstrated in Fig. 3. Ghrelin levels at GHD group increased after 30, 60, and 120 min of the CSACS, while decreased in NGH group. GH levels decreased at 30 min and significantly increased at 60 and 120 min compared to baseline in the GHD group (Fig. 3a), while in the NGH participants, increased GH could be observed at 30 and 60 min compared with the previous time interval and then GH secretion decreased a little (Fig. 3b). GH level was higher in the NGH group at 30, 60, and 120 min compared with the GHD group. It was worth noting that the serum levels of Ghrelin and GH in GHD group showed a clear correlation.

### Arginine and clonidine treatment could accelerate the height SDS for bone age

**Fig. 4:**
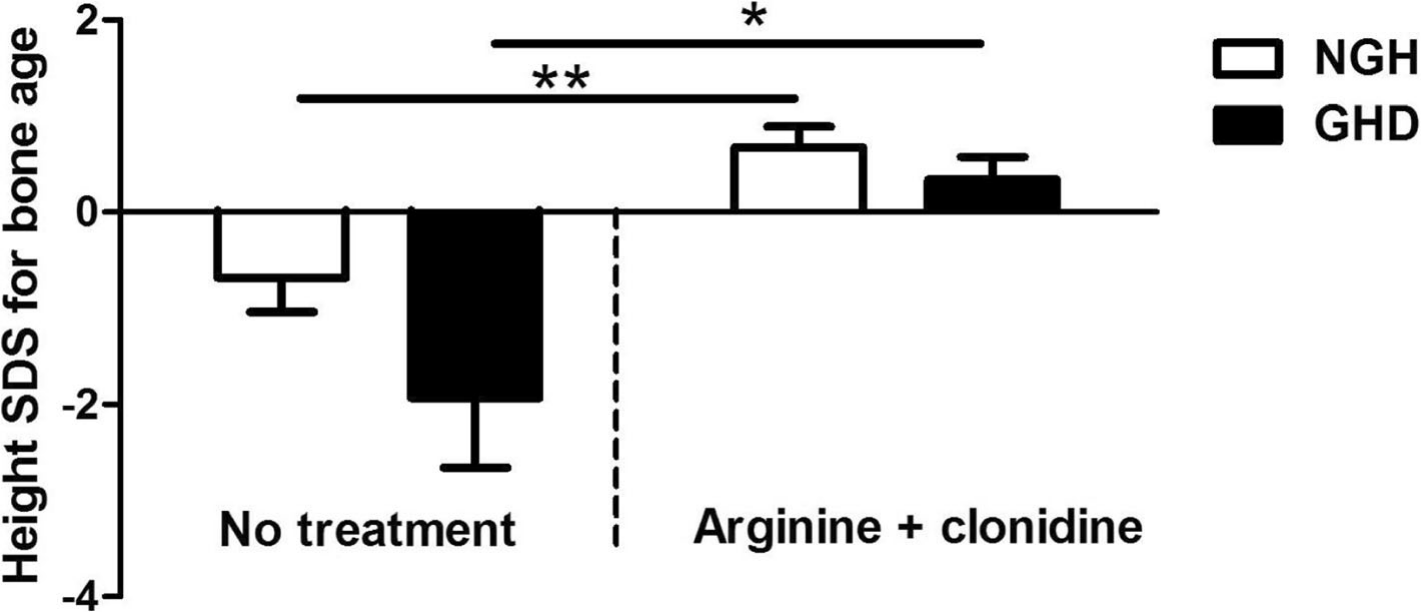
Change in height standard deviation score (SDS) for bone age between NGH and GHD groups after 6 months arginine and clonidine treatment

The 6 months CSACS treatment could increase the height SDS for bone age in both NGH and GHD groups which could be used as a measure of growth prognosis. The decreasing negative scores after the stimulation test indicated that the growth acceleration of children in NGH and GHD groups were gradually increased and got closer to the average height (Fig. 4).

## Discussion

Dynamic detection shows that CSACS can induce different Ghrelin and GH responses pattern in the GHD and NGH groups. Compared with the NGH participants, Ghrelin levels in the GHD group are higher at 30, 60, and 120 min after CSACS test, while the GH peak in GHD group is lower than NGH group. Circulating Ghrelin and GH show an inverse correlation prior to the GH peak in NGH group; while in the GHD group, an increase in serum GH levels is followed by the accelerating circulating Ghrelin. The dynamic change pattern of Ghrelin and GH in the GHD and NGH groups are difficult to interpret for these subjects involved may have different genetic traits [14, 15] and the participants sample recruited is relative small [16, 17]. The results of the present study need to be confirmed in future larger clinical trials and more detailed genetic information should be considered.

Although Ghrelin is not the principal stimulator of GH synthesis and secretion, it has a marked growth hormone-stimulating activity which can link gastrointestinal-pituitary axis and bind with the growth hormone secretagogue receptor to stimulate IP3 signal transduction pathway to promote GH release [18]. While in our research, the rise in circulating GH is positively correlated with Ghrelin in GHD group and negatively correlated with Ghrelin in NGH group prior to the GH peak, which indicates that Ghrelin might be inhibited by the secretion of GH provoked by CSACS and a feedback loop between GH and Ghrelin may exist.

Height growth results from the proliferation of chondrocyte and the hypertrophy of the growth plates which can mainly attribute to GH stimulation [19, 20]. IGF-1, also called somatomedin C, is a primary mediator of the effects of GH, which plays a vital role in childhood growth and adults anabolic process. Mecasermin, a synthetic analog of IGF-1, is designed to treat growth failure. Because IGF-1 secretion can maintain a constant daily level and GH level shows large diurnal fluctuation, IGF-1 was considered as surrogate measures of GH secretion [21, 22], which turns out not sensitive enough to diagnose GHD [23, 24]. Our study demonstrates some positive relationship between GH and IGF-1 in both NGH and GHD groups.

## Conclusions

In conclusion, our findings indicate that CSACS can not only be used to provoke GH secretion but also increase the height SDS for bone age in both NGH and GHD groups. The dynamic Ghrelin and GH secretion detection indicates some negative regulation or feedback mechanism lying between GH and Ghrelin. CSACS could be used as a height acceleration option, further investigations are needed to confirm the results and better decipher the relationship between Ghrelin and GH and the underlying mechanisms.

CSACS may be used to dynamically detect Ghrelin and GH and to accelerate the growth of children with dwarfism.

## Abbreviations

CSACS: Combined simultaneous arginine clonidine stimulation
GH: Growth hormone
GHD: Growth hormone deficiency
NGH: Normal growth hormone
SDS: Standard deviation score
